# Early coagulation tests predict risk stratification and prognosis of COVID-19

**DOI:** 10.18632/aging.103581

**Published:** 2020-08-29

**Authors:** Lili Luo, Min Xu, Mengyi Du, Haiming Kou, Danying Liao, Zhipeng Cheng, Heng Mei, Yu Hu

**Affiliations:** 1 Institute of Hematology, Union Hospital, Tongji Medical College, Huazhong University of Science and Technology, Wuhan 430022, China; 2 Hubei Clinical and Research Centre of Thrombosis and Hemostasis, Wuhan 430022, China

**Keywords:** COVID-19, blood coagulation disorders, risk, prognosis, meta-analysis

## Abstract

The ongoing outbreak of Coronavirus Disease 2019 (COVID-19) is hitting the world hard, but the relationship between coagulation disorders and COVID-19 is still not clear. This study aimed to explore whether early coagulation tests can predict risk stratification and prognosis. PubMed, Web of Science, Cochrane Library, and Scopus were searched electronically for relevant research studies published up to March 24, 2020, producing 24 articles for the final inclusion. The pooled standard mean difference (SMD) of coagulation parameters at admission were calculated to determine severe and composite endpoint conditions (ICU or death) in COVID-19 patients. Meta-analyses revealed that platelet count was not statistically related to disease severity and composite endpoint; elevated D-dimer correlated positively with disease severity (SMD 0.787 (0.277-1.298), P= 0.003, I^2^= 96.7%) but had no significant statistical relationship with composite endpoints. Similarly, patients with prolonged prothrombin time (PT) had an increased risk of ICU and increased risk of death (SMD 1.338 (0.551-2.125), P = 0.001, I^2^ = 92.7%). Besides, increased fibrin degradation products (FDP) and decreased antithrombin might also mean the disease is worsening. Therefore, early coagulation tests followed by dynamic monitoring is useful for recognizing coagulation disorders accompanied by COVID-19 and guiding timely therapy to improve prognosis.

## INTRODUCTION

In December 2019, a group of patients with unexplained pneumonia in Wuhan, China was found to be infected with a previously unknown coronavirus, officially named later as Coronavirus Disease 2019 (COVID-19). The coronavirus was initially called 2019-nCoV but was subsequently renamed severe acute respiratory syndrome coronavirus 2 (SARS-Cov-2) because it has 75-80% genomic similarity to SARS-CoV and 50% resemblance to the Middle East Respiratory Syndrome coronavirus (MERS-CoV) [1]. SARS-CoV2 is the third known kind of coronavirus that causes severe acute respiratory distress syndrome (ARDS) in humans, the others being SARS-CoV and MERS-CoV. As of April 7, 2020, 1,342,184 cases have been confirmed worldwide. Although the fatality rate will continue to change until all infected persons have recovered, it appears that SARS-CoV2 is less deadly (approximately 3.7%) than SARS-CoV (~10%) and much less than MERS-CoV (~40%) [2, 3]. Regrettably, the outbreak of COVID-19 is spreading wide and amplifying mainly because of the long incubation period and high infection rates, raising great public health concerns globally.

Unfortunately, some studies have revealed that mortality rates in critical COVID-19 patients are high (~41.7%), possibly because of the association of the disease with severe complications, including organ failure, sepsis/septic shock, and sepsis-associated coagulopathy [4–11]. Generally, the three conditions mentioned above are complexly linked in critical patients. Sepsis is consistently common in severe patients with SARS-CoV2 infection as a secondary disease [5]. Septic shock and sepsis-associated coagulopathy are severe conditions of sepsis, both of which can result in organ failure. The early reported incidence of at least one organ dysfunction is about 30%~60% in critically ill patients and non-survivors [5, 6, 12, 13], while the reported incidence of shock varies from 23% to 70% [5, 6, 13]. However, coagulopathy in COVID-19 has been reported rarely; only three articles have mentioned this problem up to now.

In the first report of the occurrence of disseminated intravascular coagulation (DIC), the worst form of coagulopathy, in a large epidemiological study on COVID-19, only 0.6% of the patients with severe cases had DIC; the standard used for diagnosis was not mentioned, and no one had DIC among non-severe patients [8]. Tang’s analysis focusing on abnormal coagulation parameters revealed that 71.4% (15/21) of non-survivors with COVID-19 met the criteria for overt-DIC [11]. Zhou and his colleagues later found that 50% of non-survivors with COVID-19 had coagulopathy, and only 7% of survivors had coagulopathy [5]. However, DIC encompasses a broad spectrum of clinical manifestations, ranging from a prothrombotic state to bleeding or both [14], and there is a lack of a golden approach to diagnosing DIC, easily leading to misdiagnosis and missed diagnoses. To optimize patient care and resource allocation during this pandemic, coagulation parameters reflecting coagulopathy and DIC are urgently needed for risk stratification and for actively monitoring illness severity.

Abnormal coagulation parameters reflecting coagulopathy, including platelet count, D-dimer level, prothrombin time (PT), and activated partial thromboplastin time (APTT), are common in many COVID-19 patients at admission. However, these indicators, as presented in different articles, are providing contradictory messages to guiding risk stratification and predicting outcomes. Although two independent teams have shown that severe COVID-19 patients have significantly lower platelet counts than non-severe patients [10, 15], other teams have demonstrated that there is no significant difference between the two groups [6, 7, 13, 16–18]. Almost all related articles have reported that critical or non-survivor patients had statistically significantly higher levels of D-dimer than non-severe or survivor patients [4, 6, 10, 19–22], except for one [15]. PT is more prolonged in severe patients in some articles [6, 10, 11], but not so in other reports [4, 13, 19, 23]. APTT in severe COVID-19 patients appears more complicated, longer than in non-severe patients [10] or shorter than in non-severe patients [4, 21] or similar to the one in non-severe patients [6, 11, 13, 23, 24]. Some reports have shown that there is no significant difference in fibrinogen levels between severe COVID-19 patients and non-severe patients [11, 17, 19], but one article found higher levels in severe patients [23]. Therefore, we did a meta-analysis and a systematic review to comprehensively analyze the significance of early coagulation tests and understand coagulopathy during COVID-19 progression for disease stratification and prediction of the composite endpoint (ICU admission or death).

## RESULTS

### The outcome of the electronic search

**Figure 1:**
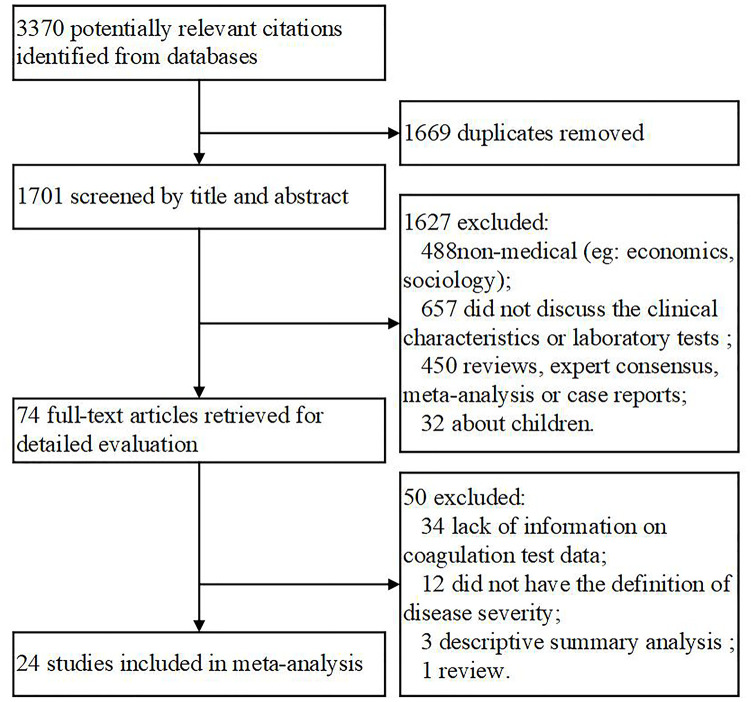
**Flow chart of the included studies.**

Overall, 3370 documents were initially identified based on our search criteria and a reference list (Figure 1). Subsequently, 1669 files were excluded because of duplication, and 1627 were excluded after reading the title and abstract and finding that the materials were not related to medicine (n = 488) or failed to report clinical characteristics or laboratory tests (n = 657) or that they were reviews (n = 271), or expert consensus (n = 96), meta-analyses (n = 9), or case reports (n = 74). Additionally, 32 documents relating to children were excluded. As a result, 74 articles were selected for full-text assessment. Of the 74 studies, 50 were disqualified for lacking information on coagulation test data (n = 34), or having no definition of disease severity (n = 12), or lacking descriptive summary analyses (n = 3), or being a review (n = 1). In the end, 24 articles were included for the meta-analysis. To eliminate bias, the detailed endpoint was split into severity and composite endpoint instead of a rough poor outcome. Also, we analyzed several biomarkers individually rather than treat them as one entity.

### Characteristics of the 24 selected studies

Of the articles included, 23 were full-length articles published in peer-reviewed journals, and one article was provided by the corresponding author after we reached out to them. Most of the studies were from China (n = 22), except for two from Singapore. All the investigations were case-control trials assessing 3544 adult COVID-19 patients; the sample size of each study varied from 21 to 1099 participants. The vast majority of patients were diagnosed using laboratory nucleic acid tests, except for three patients who were diagnosed based on clinical characteristics and imaging data. The details of the selected studies are provided in Table 1.

**Table 1:** Basic information of included studies.

**Num**	**Study cohort**	**Journal**	**Institute/region**	**Period**	**Follow-up**	**Study type**	**No.(M/F)**	**Diagnose**	**Age (year)**	**Compared endpoint**	**NOS**
1	Cao B ^6^	Lancet	Jinyintan Hospital & Wuhan Pulmonary Hospital	2019/12/29-2020/1/31	NA	case control	191 (119/72)	laboratory-confirmed	56.0 (46.0–67.0)	composite endpoint	8
2	Sun ZY ^11^	J. Thromb. Hemost.	Tongji Hospital	2020/1/1 - 2020/2/3	2020/2/13	case control	183 (98/85)	laboratory-confirmed	54.1 (14-94)	composite endpoint	8
3	Cao B (2) ^5^	Lancet	Jinyintan Hospital	2019/12/16 -2020/1/2	2020/1/22	case control	41 (30/11)	laboratory-confirmed	49.0 (41·0-58.0)	composite endpoint	8
4	Ning Q ^21^	NA	Tongji Hospital	2019/12/19-2020/1/27	2020/2/2	case control	21 (17/4)	laboratory-confirmed	56.3 (42.0-70.6)	severity status	8
5	Peng ZY ^13^	JAMA	Zhongnan Hospital	2020/1/1- 2020/1/28	2020/2/3	case control	138 (75/63)	laboratory-confirmed	56 (42-68)	composite endpoint	8
6	Zhong NS ^8^	NA	552 hospitals	2020/1/29	2020/1/29	case control	1099 (640/459)	laboratory-confirmed	47.0 (35.0-58.0)	severity status/composite endpoint	8
7	Song YL ^2^	JAMA Internal Medicine	Jinyintan Hospital	2019/12/24- 2020/1/26	2020/2/13	case control	201 (128/73)	laboratory-confirmed	51(43-60)	severity status/composite endpoint	8
8	Hu B ^22^	NA	Union Hospital	2020/1/16- 2020/2/19	NA	case control	214 (127/87)	laboratory-confirmed	52.7 (37.2-68.2)	severity status	8
9	Zhang YX ^15^	Clin Infect Dis	Zhongnan Hospital	2020/1/1-2020/2/5	NA	case control	155 (86/69)	laboratory-confirmed	54 (42-66)	severity status	8
10	Li LJ ^36^	BMJ	Zhejiang Province	2020/1/10-2020/1/26	2020/1/26	case control	62 (36/27)	laboratory-confirmed	41 (32-52)	severity status	8
11	Shang Y ^12^	The Lancet Respiratory Medicine	Jinyintan Hospital	2019/12-2020/1/26	2020/2/9	case control	52 (35/17)	laboratory-confirmed	59.7 (46.4-73.0)	composite endpoint	8
12	Ong, K H ^16^	Am J Hematol	Singapore	2020/1/23-2020/2/28	2020/2/28	case control	67 (37/30)	laboratory-confirmed	42(35-54)	composite endpoint	8
13	Wang Q ^18^	Journal of medical virology	Huizhou municipal central hospital from	2020/1-2020/2	2020/2/21	case control	30 (16/14)	laboratory-confirmed	50.5 (36-65)	severity status	8
14	Hu Y ^27^	Chin Med J	Tongji Hospital	2019/12/30-2020/1/15	2019/12/30-2020/1/15	case control	78 (39/39)	laboratory-confirmed	38 (33-57)	severity status	8
15	Chen XM ^17^	QJM	Zhejiang province	2020/1/20-2020/2/11	2020/2/16	case control	91 (37/54)	88 laboratory-confirmed & 3 clinical-confirmed	50 (36.5-57)	severity status	8
16	Gao YD ^20^	Allergy	No. 7 Hospital of Wuhan	2020/1/16-2020/2/3	NA	case control	140 (71/69)	laboratory-confirmed	57 (25-87)	severity status	8
17	Zhang RG ^7^	Clin Infect Dis	Union Hospital	2020/1/16-2020/1/29	2020/2/4	case control	69 (32/37)	laboratory-confirmed	42.0 (35.0-62.0)	severity status	8
18	Zhu CL ^19^	Clinical chemistry and laboratory medicine	Renmin Hospital	2020/1/31-2020/2/10	NA	case control	134 (76/68)	laboratory-confirmed	NA	severity status	9
19	Wang LD ^23^	Journal of medical virology	Fuyang Second people's hospital	2020/1/23-20202/2	NA	case control	43 (26/17)	laboratory-confirmed	43.74 ± 12.12	severity status	8
20	Zeng QT ^24^	Zhonghua xin xue guan bing za zhi	Union Hospital	2020/1/20-2020/2/15	NA	case control	112 (53/59)	NA	62 (55-67)	severity status	8
21	Li CH ^28^	Chinese journal of tuberculosis and respiratory diseases	Jianghan university hospital	2020/1/10-2020/1/31	NA	case control	30 (10/20)	laboratory-confirmed	35(27-43)	severity status	8
22	Barnaby EY ^26^	JAMA	Singapore	2020/1/23-2020/2/3	2020/2/25	case control	18 (9/9)	laboratory-confirmed	47 (31-73)	severity status	8
23	Yang SR ^10^	J Med Virol	Chongqing Three Gorges Central Hospital	2020/1/23-2020/2/8	NA	case control	135 (72/63)	laboratory-confirmed	47 (36-55)	severity status	8
24	Hu Y	NA	3 designated hospitals in Wuhan	2020/1/15-2020/2/15	2020/3/10	case control	380 (207/173)	laboratory-confirmed	64 (53-73)	severity status/composite endpoint	8

The second column is the corresponding author of the article. Composite endpoint means ICU or death. Not applicable (NA); M/F (male/female); Newcastle-Ottawa quality assessment scale (NOS).

Ottawa quality assessment scale (NOS) was used to evaluate the quality of the chosen literature, and all literature scored ≥ 8 points (Supplementary Table 1), indicating that the quality of each of the 24 studies was high.

### The relationship between platelets and disease severity or composite endpoint

**Figure 2:**
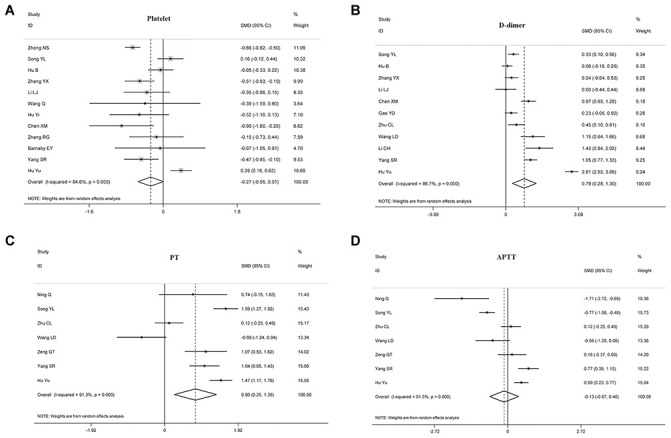
**Forest plots assessing the severity status of COVID-19 patients, as determined using coagulation parameters.** The sizes of the blocks or diamonds represent the weights, and the lengths of the straight lines represent the widths of the 95% CIs. (**A**) comparing patients by platelet counts; (**B**) comparing patients by D-dimer levels; (**C**) comparing patients by PT; (**D**) comparing patients by APTT. prothrombin time (PT); activated partial thromboplastin time (APTT).

**Figure 3:**
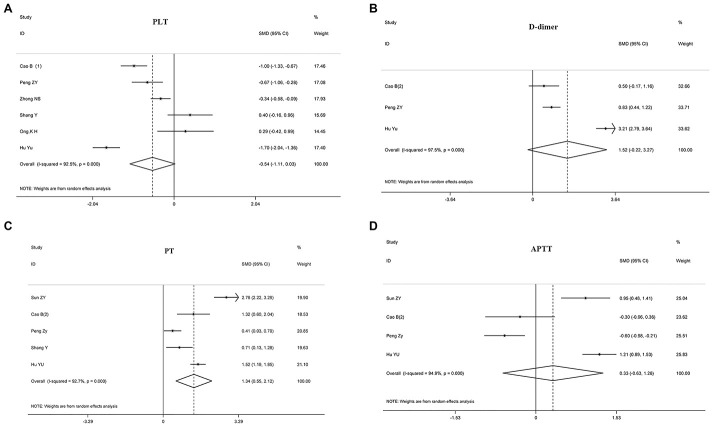
**Forest plots assessing the composite endpoint of COVID-19 patients, as determined using coagulation parameters. The sizes of the blocks or diamonds represent the weights, and the lengths of the straight lines represent the widths of the 95% CIs.** (**A**) Comparing patients by platelet counts; (**B**) comparing patients by D-dimer levels; (**C**) comparing patients by PT; (**D**) comparing patients by APTT. prothrombin time (PT); activated partial thromboplastin time (APTT).

The relationship between disease prognosis and platelets was analyzed in 16 articles with 2980 COVID-19 patients (Table 2). Of the 16 articles, 12 studies with 2152 patients were used to analyze the relationship between platelets and disease severity, [7, 8, 10, 12, 15, 17, 18, 22, 25–27] and 1778 patients in 6 articles were used to analyze the relationship between platelets and composite endpoint [6, 8, 12, 13, 16]. Pooled analyses revealed that platelet count was not statistically linked to disease severity (standard mean difference (SMD) -0.271 (-0.547-0.005), *P* = 0.054, *I^2^* = 84.6%) and composite endpoint (SMD -0.541 (-1.109-0.028), *P* = 0.062, *I^2^* = 92.5%) on admission (Table 2, Figures 2 and 3). Because the heterogeneity value was over 50%, the random effect model was used for the meta-analysis of these articles.

**Table 2:** Summary of the meta-analysis results.

**Biomarker**	**Total no. of studies**	**Total no. of patients**	**Endpoint**	**No. of studies**	**No. of patients**	**Statistical method**	**pooled Standard Mean Difference (SMD)**	**P**	**I^2^**	**P (Heterogeneity)**	**P Begg’s Test**	**P Egger’s test**
Platelet	16	2980	severity status	12	2152	I-V, Random	-0.271 (-0.547-0.005)	0.054	84.60%	<0.001	0.732	0.951
			composite endpoint	6	1778	I-V, Random	-0.541 (-1.109-0.028)	0.062	92.50%	<0.001	0.462	0.413
PT	11	1641	severity status	7	940	I-V, Random	0.803 (0.254-1.352)	**0.004**	91.30%	<0.001	0.368	0.224
			composite endpoint	5	645	I-V, Random	1.338 (0.551-2.125)	**0.001**	92.70%	<0.001	1.000	0.300
APTT	10	1388	severity status	7	940	I-V, Random	-0.133 (-0.668-0.402)	0.625	91.50%	<0.001	0.368	0.499
			composite endpoint	4	593	I-V, Random	0.327 (-0.630-1.285)	0.503	94.90%	<0.001	0.734	0.591
D-dimer	13	1762	severity status	11	1438	I-V, Random	0.787 (0.277-1.298)	**0.003**	96.70%	<0.001	0.062	0.510
			composite endpoint	3	410	I-V, Random	1.523 (-0.221-3.267)	0.087	97.50%	<0.001	1.000	0.805
Fibrinogen	5	682	-	-	-	I-V, Random	0.559 (-0.599-1.718)	0.344	96.70%	<0.001	0.806	0.317
FDP	3	548	-	-	-	I-V, Random	1.046 (0.371-1.722)	**0.002**	88.90%	<0.001	1.000	0.806
Antithrombin	3	548	-	-	-	I-V, Random	-0.798(-1.217 - -0.379)	**<0.001**	72.20%	0.027	0.296	0.190			

prothrombin time (PT); activated partial thromboplastin time (APTT); fibrin/fibrinogen degradation products (FDP).

### The relationship between D-dimer and disease severity or composite endpoint

In this meta-analysis, we explored the relationship between D-dimer and prognosis in 1762 patients with COVID-19 from 13 investigations (Table 2). Based on the data from 1438 participants in 11 trials, [4, 10, 15, 17, 19, 20, 22, 23, 25, 28].

We found that D-dimer correlated positively with disease severity in patients with COVID-19 (SMD 0.787 (0.277-1.298), *P* = 0.003, *I^2^* = 96.7%), suggesting that D-dimer levels were significantly elevated in critically ill patients. Also, 410 patients in three articles were assessed for the relationship between D-dimer and composite endpoint [5, 13], but we found no statistical relationship between the two parameters (SMD 1.523 (-0.221-3.267), *P* = 0.0087, *I^2^* = 97.5%), see Table 2, Figures 2 and 3.

### The relationship between PT and disease severity or composite endpoint

Eleven articles with 1641 patients were analyzed for PT; 7 articles with 940 cases were evaluated for the relationship between PT and disease severity [4, 10, 19, 21, 23], and 5 articles with 645 cases were examined for the relationship between PT and composite endpoint [5, 11–13]. The analyses showed that prolonged PT during admission indicated a more serious disease, with the two correlating positively (SMD 0.803 (0.254-1.352), *P* = 0.004, *I^2^* = 91.3%). Similarly, patients with prolonged PT had an increased risk of ICU during admission and increased risk of death (SMD 1.338 (0.551-2.125), *P* = 0.001, *I^2^* = 92.7%), see Table 2, Figures 2 and 3.

### The relationship between APTT and disease severity or composite endpoint

10 articles with 1388 COVID-19 patients were analyzed for the relationship between disease prognosis and APTT (Table 2); 7 articles with 940 patients were assessed for the relationship between APTT and disease severity [4, 10, 19, 21, 23, 24], and 593 patients in four articles were studied for the relationship between APTT and composite endpoint [5, 11, 13]. Our results revealed that APTT was not statistically associated with disease severity and composite endpoint at admission (Table 2, Figures 2 and 3).

### The relationship between fibrinogen, fibrin/fibrinogen degradation products (FDP), antithrombin, and prognosis

Five studies with 682 patients were analyzed for the effect of fibrinogen on prognosis [11, 17, 19, 23]. We found that fibrinogen had no value in predicting disease prognosis in COVID-19 patients (SMD 0.559 (-0.599-1.718), *P* = 0.344, *I^2^* = 96.7%) (Supplementary Figure 1). Furthermore, 548 cases in three articles were evaluated for the relationship between FDP, antithrombin, and prognosis [11, 19]. Our results revealed that increased FDP (SMD 1.046 (0.371-1.722, *P* = 0.002, *I^2^* = 88.9%) and decreased antithrombin (SMD -0.798 (-1.217-0.379), *P*<0.001, *I^2^* = 72.2%) were associated with the worsening of COVID-19 (Table 2, Supplementary Figure 2).

### Sensitivity analysis and publication bias

A Funnel plot was drawn to test publication bias, and Egger's test and Begg’s test indicated that there was no publication bias (Supplementary Figures 3, 4). Sensitivity analysis revealed that no study greatly interfered with the results of this meta-analysis study greatly interfered with the results of this meta-analysis, suggesting that the study was stable (Supplementary Figures 5, 6).

## DISCUSSION

COVID-19 has raised great public health concerns globally over the last three months. Like with SARS, abnormal coagulation disorders are common in severe patients with COVID-19. Our meta-analysis combined the outcomes of 3544 COVID-19 patients from 24 separate studies and established that elevated D-dimer significantly predicted more severe classifications of COVID-19 patients. Prolonged PT at baseline also suggested poor outcomes, both in severity status and composite endpoint. Increased FDPs and decreased antithrombin might also signal severe conditions.

The platelet count at admission had no remarkable relationship with outcome. However, a meta-analysis involving 399 subjects showed that platelet counts at admission were significantly lower in more severe and non-survivor COVID-19 patients [29]. This discrepancy in outcome regarding platelet counts may be due to inconsistencies in the selected literature. A national multi-center retrospective study led by Academician Zhong supported the conclusion that platelet count is not statistically linked to a composite endpoint, although the authors also found that severe patients had lower platelets on admission than non-severe patients. One possible reason was the difference in the research objects. In other selected articles, the patients were either in Wuhan or outside Wuhan. The objects in Zhong’s article included hospitalized patients both in Wuhan and outside-Wuhan. The early epidemic situation in Wuhan was overwhelming, medical resources were tight, and patients with a milder disease were isolated at home while more severe patients were admitted to the hospital. Patients hospitalized outside-Wuhan got sufficient resources due to they having relatively few cases at the time.

Platelets play a crucial role in hemostasis and thrombosis. While platelet activation and thrombocytosis increase the risk of thrombotic complications, platelet function disorders and thrombocytopenia increase bleeding risk. Thrombocytopenia and reactive thrombocytosis are both common in a variety of viral infections [30–35]. During SARS, most patients’ platelet counts were normal at the onset of the disease, but, with time, 55% developed thrombocytopenia (platelet count < 140×10^9^/L), and 49% harbored reactive thrombocytosis (platelet count ≥ 400×10^9^/L) [32]. Similarly, in COVID-19 patients, platelet counts were also within the normal range in most cases at admission [4, 7, 13, 15, 16, 26, 36]; thrombocytopenia (platelet count < 100 ×10^9^/L) was reported primarily in severe patients or non-survivors (20%~66.1%) [5, 8, 11], while thrombocytosis was reported in a few articles, and the proportion was not assessed [21]. The outcome of platelet count changes for the entirety of COVID-19 infection in patients has rarely been reported. Until recently, according to the article with 1476 COVID-19 patients by Yang et al., platelet counts in survivors tended to be stable during hospitalization, but they progressively decreased in non-survivors [37]. Furthermore, the lower the nadir platelet count during hospitalization, the higher the risk of death [37].

Thrombocytopenia is often considered an indicator of bleeding and mortality in critical patients [38]. Decreased platelet counts help recognize the presence and severity of coagulopathy [39]. The mechanisms of thrombocytopenia during COVID-19 might include direct or indirect factors induced by the SARS-Cov2 infection, such as inappropriate platelet activation and consumption, immunological platelet destruction, and impaired megakaryopoiesis [40]. Recently, Levi M et al. proposed that localized pulmonary thrombotic microangiopathy where platelet consumption is a common feature, may partly account for thrombocytopenia [41]. Additionally, two independent teams found that COVID-19 patients in ICU had markedly elevated levels of the von Willebrand factor [42, 43], further supporting Levi M’s opinion. Though COVID-19-associated coagulopathy belongs to sepsis-induced coagulopathy, thrombocytopenia is less profound [43], which may be related with that COVID-19-accociated-coagulopathy was a severe hypercoagulability rather than consumptive coagulopathy [44]. Bleeding events are less documented or reported in current articles looking at the clinical features of COVID-19, although autopsies have revealed focal hemorrhage in the lungs and spleen and decreased myelopoiesis in the bone marrow [45]. Mao’s team found that one of 88 severe patients had a cerebral hemorrhage [21]. Yang et al. showed that 6% of 32 non-survivors had a gastrointestinal hemorrhage [12]. In addition to low platelets, bleeding events in critical COVID-19 patients may also be linked to corticosteroid therapy in more critically ill patients. In the interim guidance of coagulopathy in COVID-19, the ISTH recommends that platelet counts be kept above 50×10^9^/L in bleeding patients and above 20×10^9^/L in non-bleeding patients.

D-dimer, a more specific marker than FDP reflecting the dissolution of microthrombi, is amplified in septic patients [46], consistent with what is reported in COVID-19 patients [6, 10, 11, 20, 22, 23]. In non-COVID-19 septic patients, D-dimer concentrations do not reach the high values seen in patients with COVID-19 [41, 43]. Generally, FDP correlates positively well with D-dimer, except in some situations, like primary hyperfibrinolysis, and simultaneous measurements of FDPs and D-dimer are useful for more accurate estimations of fibrinolytic states [47]. However, of the articles that met our inclusion criteria, only three provided FDP information, whereas, many articles recorded D-dimer changes. Strikingly, 43.2%~68% of COVID-19 patients had elevated levels of D-dimer [5, 8, 20], and this proportion was as high as 92% in dead patients [5]. Increased D-dimer levels generally indicate a high risk of thrombotic diseases [48]. By the time we started this meta-analysis, the incidence of thrombosis had rarely been reported in COVID-19 patients, although thrombosis and microthrombosis in multiple organs had been observed during autopsies [45]. In a study specifically looking at neurological manifestations, Mao and colleagues revealed that 4.5% of severe COVID-19 patients had an acute ischemic stroke [22]. Another study found that 3.4% of severe COVID-19 patients had a stroke [20]. Recently, several teams in different countries emphasized the high incidence of thrombotic events in severe COVID-19 patients. In a study of 81 ICU patients without routine thromboprophylaxis in China, the incidence of deep vein thrombosis was 25% [49]. In Netherlands, two independent researches where routine low molecular weight heparin prophylaxis was applied, reported similar (even higher) incidence of venous thromboembolism (VTE) among ICU patients with COVID-19 [50, 51]. Most recently, Helms et al. showed that COVID-19-ARDS patients developed significantly more thrombotic complications than non-COVID-19-ARDS patients based on a multicenter prospective cohort study [43].

In COVID-19 patients, especially severe patients, the mechanisms of elevated D-dimer or thrombosis may include older age, chronic diseases, hypoxemia, hypercytokinemia, coagulopathy, and inevitable prolonged bed rest. It is already well-established that older individuals and those who have co-morbidities and hypercytokinemia are more likely to die from COVID-19 infection [4, 5, 7, 8, 11, 20, 21, 23, 24]. Aging and chronic diseases are recognized risk factors for sepsis, which is characterized by excessive inflammation, including hypercytokinemia and endothelial dysfunction, resulting in a hypercoagulability state [42, 52]. Refractory hypoxemia may lead to vasoconstriction reducing blood flow and promoting vascular occlusion [53]. SARS- and COVID-19-associated coagulopathy is sepsis-induced, generally characterized by markedly increased levels of plasminogen activator inhibitor-1 (PAI-1) [46, 54]. Consistently, the PAI-1 level in SARS patients is significantly higher, not only compared to healthy controls but also patients with other cases of pneumonia [55], and whether this is so in COVID-19 patients is a matter still to be verified.

Generally, coagulation tests are prolonged when the level of coagulation factors is below 50%, and an abnormality may occur up to the decompensation period of DIC because of the consumption of clotting factors during DIC progression [46, 56]. However, at the early stage of septic DIC, coagulation tests may be shortened because of hypercoagulability. This meta-analysis showed that PT, but not APTT, had an increased risk of ICU and death on admission, perhaps because coagulopathy in COVID-19 is sepsis-induced, where mostly the exogenous, but not the endogenous, coagulation pathway is activated. Given that PT and APTT are within the reference ranges on admission in most COVID-19 patients, baseline PT and APTT have limited values for risk stratification and prognosis in COVID-19 patients [5, 13, 19]. However, PT can progressively extend in nonsurvivors [11], due to the continuous activation and consumption of the exogenous coagulation pathway. As an acute reactive protein, hyperfibrinogenemia is common in the early phase of COVID-19 in both survivors and non-survivors [11]. Yet, the level of fibrinogen can progressively decrease in non-survivors, and hypofibrinogenemia may be observed at the late stage of consumption coagulopathy [11]. Antithrombin may be readily exhausted during continuous thrombin generation, with low levels of antithrombin found in approximately 50% of critically ill patients and 90% of DIC patients [56]. Therefore, the dynamic monitoring of these coagulation tests is highly recommended.

The combination of thrombocytopenia, increased D-dimer, prolonged PT, and decreased antithrombin is suggestive of DIC, though the majority of COVID-19 patients would not meet the Overt-DIC criteria established by the International Society on Thrombosis and Hemostasis (ISTH) [41, 43]. The ISTH positively recommends anticoagulants when septic patients meet the diagnostic criteria of sepsis-induced coagulopathy (SIC) [54], which could result in a significant reduction in mortality [57, 58]. However, patients with advanced coagulopathy may have a disease progression that is no longer amenable to anticoagulant therapy [59]. For that reason, the ISTH recommends a two-step diagnosis for sepsis-associated coagulopathy and emphasizes that therapeutic doses of heparin should be considered in coagulopathic patients to avoid progression from coagulopathy to DIC [54]. Increasing evidence demonstrates that there is a high risk of thrombotic complications in severe COVID-19 patients, and early anticoagulation therapy seems to improve the outcome of severe COVID-19 patients [43, 49–51, 60–62]. Tang’s team specifically looking at anticoagulant treatments showed that the 28-day mortality rate of COVID-19 patients using heparin was lower than that of nonusers in cases of severe COVID-19 patients meeting SIC criteria or with D-dimer > 3.0 ug/mL [60]. Llitjos et al. revealed that, among the twenty-six COVID-19 patients with mechanical ventilation, the incidence of VTE in patients treated with prophylactic anticoagulation was significantly higher than that in the group receiving therapeutic anticoagulation [63]. In a prospective observational study with sixteen ICU COVID-19 patients, Ranucci et al. showed that the pro-coagulant situation of patients gradually improved after thromboprophylaxis was increased [64]. Zhang et al. revealed that the thromboprophylaxis halved the incidence of DVT in COVID-19 patients with a Padua prediction score≥4 [65]. Given that COVID-19-ARDS patients had higher risk of thrombotic complications than non-COVID-19-ARDS patients, Helms et al. suggested the presence of higher anticoagulation targets in critically ill patients than usual [43]. However, the efficacy of anticoagulant therapy needs to be verified in high-quality RCT experiments. Clinicians should closely monitor indicators during the laboratory examination of patients to stay alert for side effects after anticoagulant treatment [66].

Our study has several limitations. First, all the studies included in this meta-analysis are retrospective studies with large heterogeneity. Second, the data came mainly from China; factors such as virus strain types, medical levels, countries, races, etc., may affect the results. However, at the moment, more detailed subgroup analyses cannot be conducted to comprehensively understand COVID-19 because the material for this is limited. Third, for some parameters, the number of studies included in the meta-analysis was less than 10. In this case, the publication bias may, therefore, not have been detected by Egger’s and Begg’s tests because of the relatively lower power. Fourth, the pooled sample sizes were not large enough. Precise estimates of these parameters should be assessed further.

## CONCLUSIONS

Elevated D-dimer and FDP, prolonged PT, and decreased antithrombin predict higher risk stratification and poorer prognosis in COVID-19, which is perhaps not fully in line with the facts because the studies selected for the meta-analysis were limited. There is, however, no doubt that early coagulation tests and dynamically monitoring coagulation indicators during hospitalization are helpful in the early identification of coagulation disorders, and the rational use of these parameters and the scoring systems help guide treatment and improve the prognosis of COVID-19.

## MATERIALS AND METHODS

### Search strategy

We conducted this systematic review and meta-analysis using a predefined protocol under PRISMA guidelines [67]. We searched PubMed, Web of Science, Cochrane Library, and Scopus electronically. Medical subject headings and random words (e.g. COVID-19) were combined to search the databases without language or ethnic origin restriction and dated up to March 24, 2020 (for detailed search methods, see Supplementary Table 2). The titles, abstracts, and full texts of all documents were identified independently by two investigators, and disagreements were adjudicated by a third investigator. The reference list of all identified documents was scrutinized to identify additional potentially eligible studies.

### Inclusion and exclusion

The criteria for including a study in the meta-analysis were as follows: (I) the COVID-19 patient cohort was confirmed primarily by laboratory detection; (II) the endpoint was severity status and/or composite endpoint (including ICU monitoring and death); (III) groups were established for comparison; (IV) the correlation of coagulation biomarkers with endpoints was recorded. Exclusion criteria were as follows: (I) review articles, case reports, and laboratory studies; (II) studies with insufficient data for estimating pooled standard mean differences (SMD).

### Data extraction

We collected the following items from each study, if available: the corresponding author’s name, the study type, the institute or region, the period of case collection and follow-up, the number of reported cases, disease severity, complications (e.g., coagulopathy and DIC), outcome, and laboratory findings (e.g., platelet, D-dimer, PT, APTT, or fibrinogen) were entered in a well-designed form independently by two investigators. If different articles published by the same institution overlapped during case inclusion, the research with the largest number of cases was selected, and the others were excluded. A third investigator checked the article list and data extraction to ensure that there were no duplicate articles or duplicate information and made a judgment on controversial articles.

### Quality assessment

Two reviewers independently evaluated the methodological quality of each selected study. The quality of case-control studies was evaluated using the Newcastle-Ottawa quality assessment scale (NOS) [68], which comprises 9 points; 4 points for selection, 2 points for comparability, and 3 points for the outcome. Six or more points in case-control studies were regarded as high quality. Disagreements were resolved by discussion.

### Statistical analysis

Version 12.0 of the STATA statistical software (STATA, College Station, TX) was used to calculate the combined survival impact of indicators of coagulation. The impact of biomarkers on endpoints was determined by calculating pooled mean values and their 95% CIs. Results suggested statistical significance if the 95% CI was no more than 0. Also, increased indicator levels contributed to an adverse survival effect, compared to control-patients, when the pooled mean value was more than 0. The heterogeneity of the selected studies was evaluated using the chi-squared test, with significance set at a p-value of less than 0.10. The statistic I ^2^ was used to quantify heterogeneity; an I ^2^ value less than 25% was regarded as low heterogeneity, a value between 25 and 50% indicated moderate heterogeneity, and a value over 50% signaled high heterogeneity [69]. The random-effect model was used if high heterogeneity was observed; otherwise, a fixed-effect model was used for the meta-analysis. Sensitivity analysis was applied to explore the origin of heterogeneity. Funnel plots, Begg’s test, and Egger’s test were used to screen for potential publication bias of the total population. Poor stability resulting from the inclusion and exclusion of studies was reappraised.

## Supplementary Material

Supplementary Figures

Supplementary Tables
