# Supplementary material for: Early coagulation tests predict risk stratification and prognosis of COVID-19
Source: Aging (Albany NY). 2020 Aug 29;12(16):15918–37. doi: 10.18632/aging.103581 (PMC7485702; doi:10.18632/aging.103581)
Supplement: Supplementary Figures [file aging-12-103581-s002..pdf]

SUPPLEMENTARY FIGURES

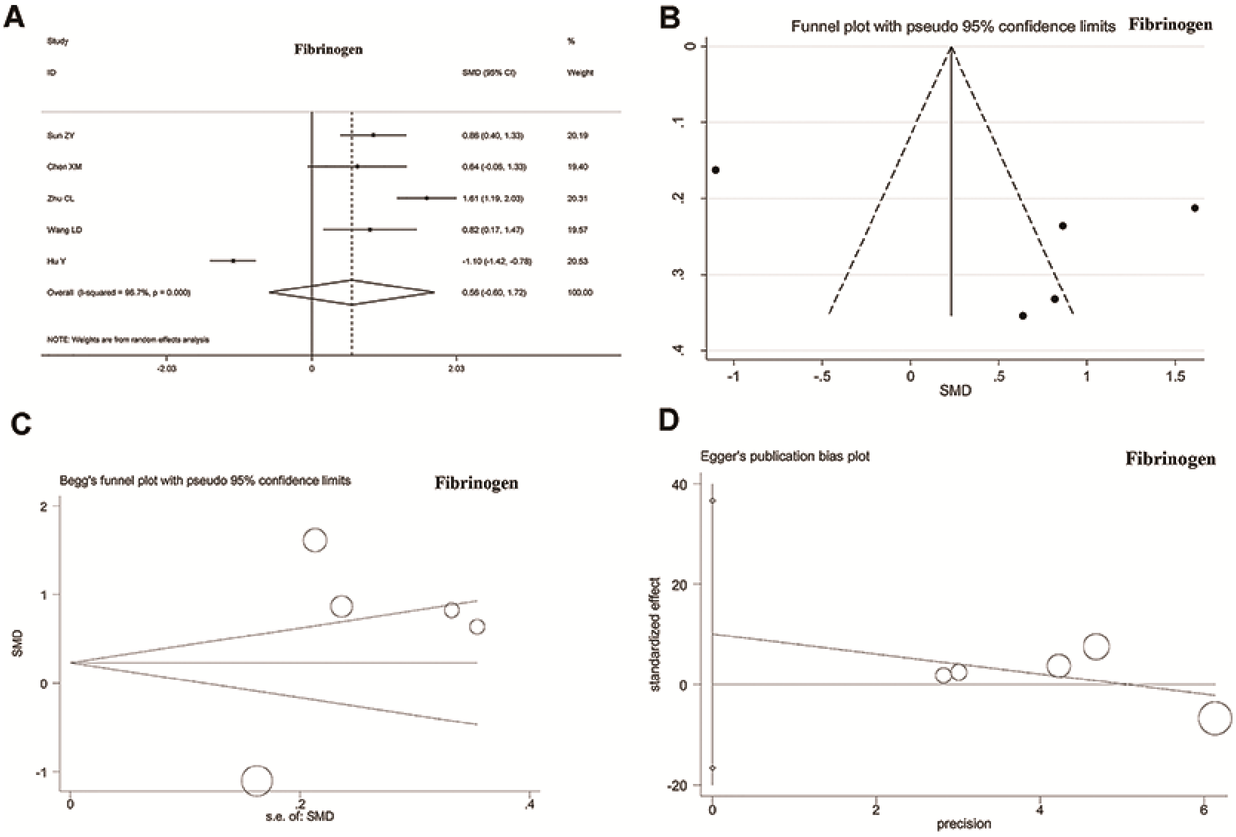

Supplementary Figure 1. Forest plots and publication bias of fibrinogen. Forest plots of pooled standard mean difference and 95% CIs assessing the severity status of COVID-19 patients by fibrinogen. The sizes of the blocks or diamonds represent the weights, and the lengths of the straight lines represent the widths of the 95% CI (**A**) Funnel plot (**B**) Egger's test (**C**) and Begg's (**D**) test assessing the publication bias of fibrinogen.

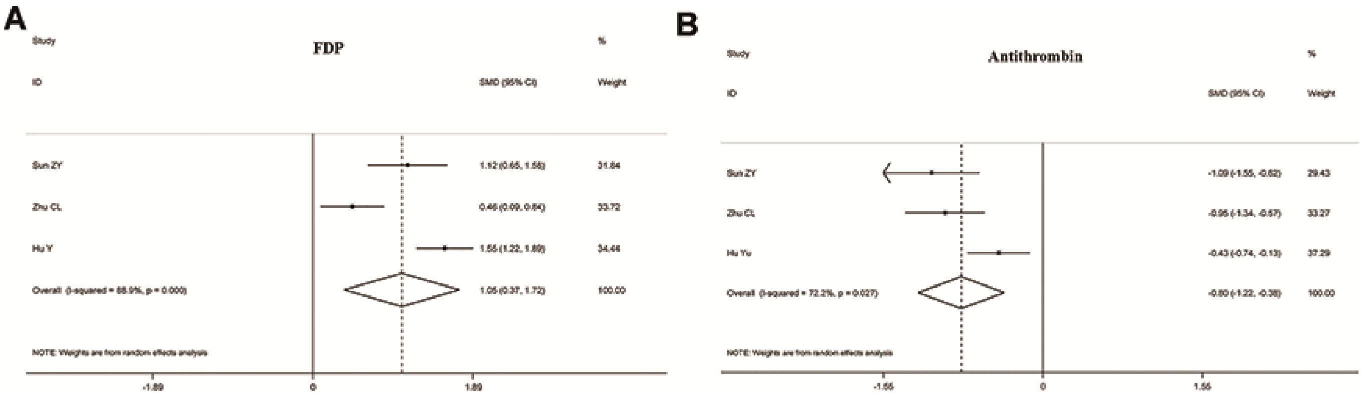

Supplementary Figure 2. Forest plots of pooled standard mean difference and 95% CIs assessing the severity status of COVID-19 patients by fibrin/fibrinogen degradation products (FDP) (**A**) and antithrombin (**B**). The sizes of the blocks or diamonds represent the weights, and the lengths of the straight lines represent the widths of the 95% CIs.

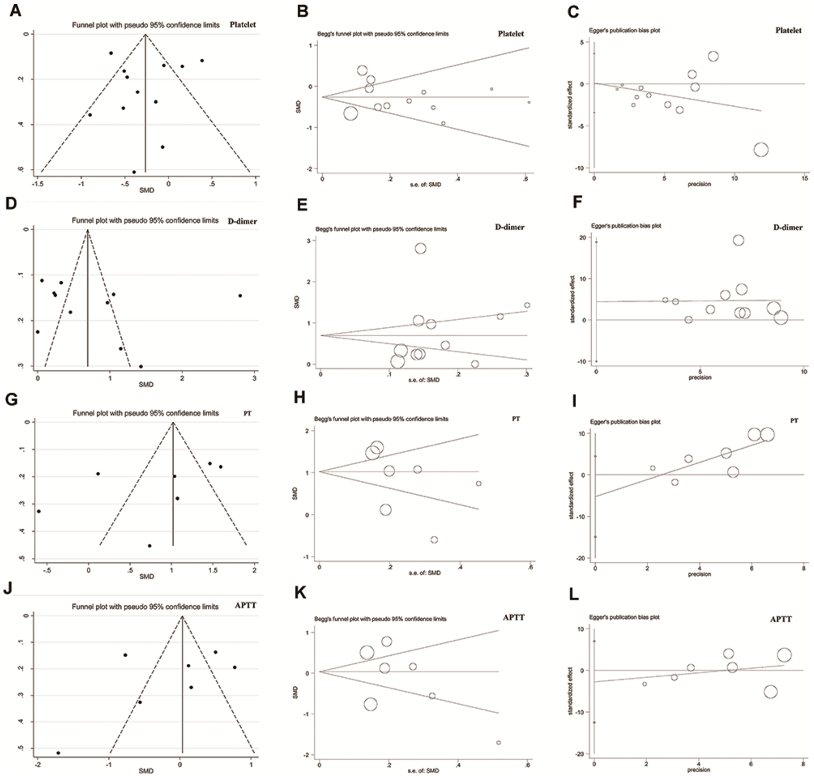

Supplementary Figure 3. Funnel plot, Egger's test and Begg's test assessing the publication bias of platelet (**A**–**C**) D-dimer (**D**–**F**) prothrombin time (PT) (**G**–**I**) and activated partial thromboplastin time (APTT) (**J**–**L**) associated with the severity status, respectively.

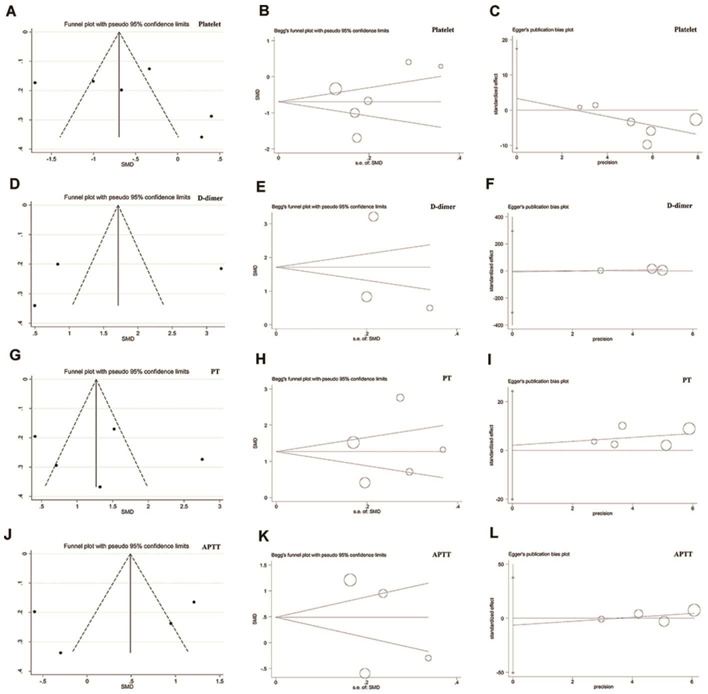

Supplementary Figure 4. Funnel plot, Egger's test and Begg's test assessing the publication bias of platelet (**A**–**C**) D-dimer (**D**–**F**) prothrombin time (PT) (**G**–**I**) and activated partial thromboplastin time (APTT) (**J**–**L**) associated with the composite endpoint, respectively.

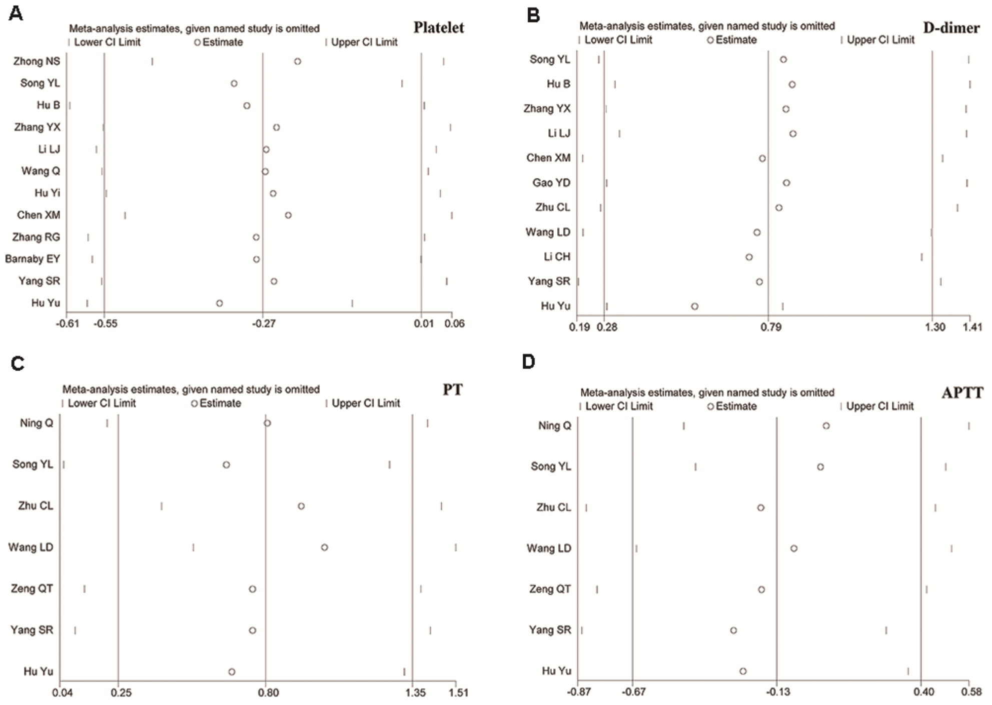

Supplementary Figure 5. Sensitivity analysis of studies involving platelet (**A**) D-dimer (**B**) prothrombin time (PT) (**C**) and activated partial thromboplastin time (APTT) (**D**) associated with the severity status. None of the articles removed would have a significant effect on the results.

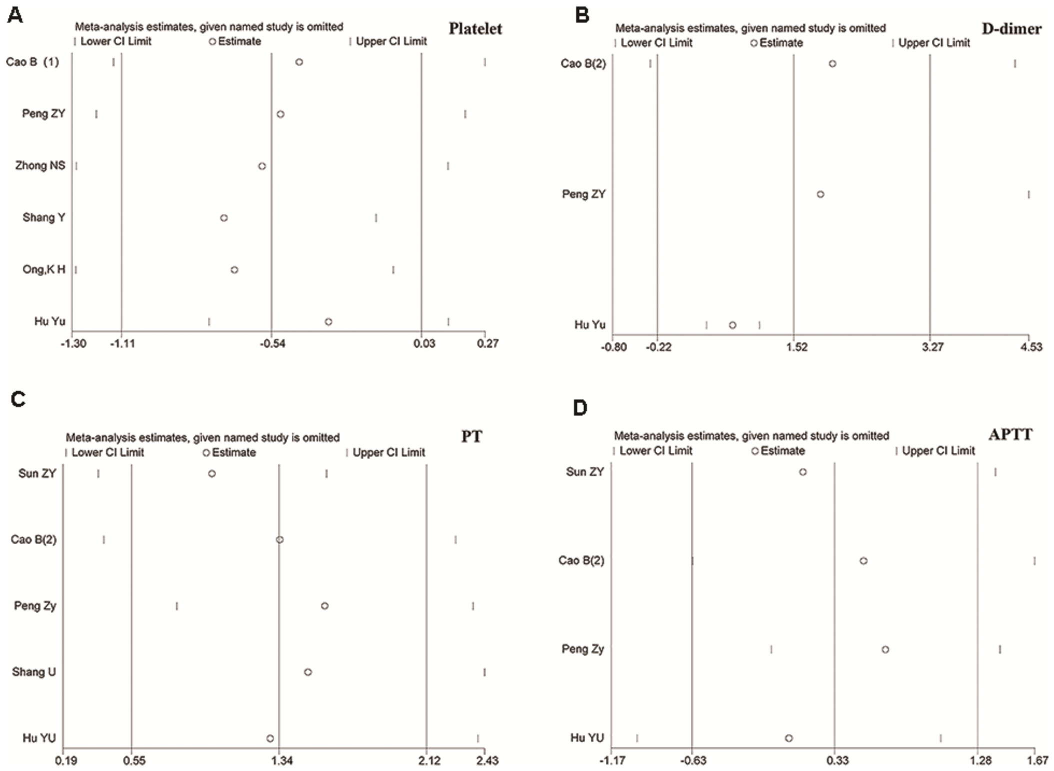

Supplementary Figure 6. Sensitivity analysis of studies involving platelet (**A**) D-dimer (**B**) prothrombin time (PT) (**C**) and activated partial thromboplastin time (APTT) (**D**) associated with the composite endpoint. None of the articles removed would have a significant effect on the results.
