# Supplementary material for: Early coagulation tests predict risk stratification and prognosis of COVID-19
Source: Aging (Albany NY). 2020 Aug 29;12(16):15918–37. doi: 10.18632/aging.103581 (PMC7485702; doi:10.18632/aging.103581)
Supplement: Supplementary Tables [file aging-12-103581-s001..pdf]

SUPPLEMENTARY TABLES

Supplementary Table 1. Newcastle-Ottawa quality assessment scale (NOS).

		Selection (****)					Exposure (***)			
NUM	Study	Case definition adequate	Representativeness of the cases	Selection of controls	Definition of controls	Comparability (**)	Ascertainment of exposure	Same method of ascertainment for cases and controls	Non-Response rate	Score
1	Cao B	*	*		*	**	*	*	*	8
2	Sun ZY	*	*		*	**	*	*	*	8
3	Cao B (2)	*	*		*	**	*	*	*	8
4	Ning Q	*	*		*	**	*	*	*	8
5	Peng ZY	*	*		*	**	*	*	*	8
6	Zhong NS	*	*		*	**	*	*	*	8
7	Song YL	*	*		*	**	*	*	*	8
8	Hu B	*	*		*	**	*	*	*	8
9	Zhang YX	*	*		*	**	*	*	*	8
10	Li LJ	*	*		*	**	*	*	*	8
11	Shang Y	*	*		*	**	*	*	*	8
12	Ong, K H	*	*		*	**	*	*	*	8
13	Wang Q	*	*		*	**	*	*	*	8
14	Hu Y	*	*		*	**	*	*	*	8
15	Chen XM	*	*		*	**	*	*	*	8
16	Gao YD	*	*		*	**	*	*	*	8
17	Zhang RG	*	*		*	**	*	*	*	8
18	Zhu CL	*	*	*	*	**	*	*	*	9
19	Wang LD	*	*		*	**	*	*	*	8
20	Zeng QT	*	*		*	**	*	*	*	8
21	Li CH	*	*		*	**	*	*	*	8
22	Barnaby EY	*	*		*	**	*	*	*	8
23	Yang SR	*	*		*	**	*	*	*	8
24	Hu Y	*	*		*	**	*	*	*	8

Supplementary Table 2. Research strategy.

PubMed	**Mesh**: severe acute respiratory syndrome coronavirus 2 COVID-19; spike glycoprotein; COVID-19 virus<br>**Entry Terms**: Wuhan coronavirus; Wuhan seafood market pneumonia virus; COVID19 virus; coronavirus disease 2019 virus; SARS-CoV-2; SARS2; 2019-nCoV; 2019 novel coronavirus; 2019 novel coronavirus infection; COVID19; coronavirus disease 2019; coronavirus disease-19; 2019-nCoV disease; 2019 novel coronavirus disease; 2019-nCoV infection; COVID-19 virus spike glycoprotein; 2019-nCoV spike glycoprotein<br>**Search** (((((((((((((((((((severe acute respiratory syndrome coronavirus 2) OR COVID-19) OR spike glycoprotein, COVID-19 virus) OR Wuhan coronavirus) OR Wuhan seafood market pneumonia virus) OR COVID19 virus) OR coronavirus disease 2019 virus) OR SARS-CoV-2) OR SARS2) OR 2019-nCoV) OR 2019 novel coronavirus) OR 2019 novel coronavirus infection) OR COVID19) OR coronavirus disease 2019) OR coronavirus disease-19) OR 2019-nCoV disease) OR 2019 novel coronavirus disease) OR 2019-nCoV infection) OR COVID-19 virus spike glycoprotein) OR 2019-nCoV spike glycoprotein
Web of Science	**TS=**(severe acute respiratory syndrome coronavirus2 OR COVID-19 OR spike glycoprotein, COVID-19 virus OR Wuhan coronavirus OR Wuhan seafood market pneumonia virus OR COVID19 virus OR coronavirus disease 2019 virus OR SARS-CoV-2 OR 2019-nCoV OR 2019 novel coronavirus OR 2019 novel coronavirus infection OR COVID19 OR coronavirus disease 2019 OR coronavirus disease-19 OR 2019-nCoV disease OR 2019 novel coronavirus disease OR 2019-nCoV infection OR COVID-19 virus spike glycoprotein OR 2019-nCoV spike glycoprotein)
Cochrane Library	We put “COVID-19” into the Mesh box, but no Mesh terms and Tree were available
Scopus	**Search** ("severe acute respiratory syndrome coronavirus2") OR (COVID-19) OR ("spike glycoprotein, COVID-19 virus") OR ("Wuhan coronavirus")OR("Wuhan seafood market pneumonia virus ")OR(" COVID19 virus") OR ("coronavirus disease 2019 virus ")OR ("SARS-CoV-2") OR ("2019-nCoV ")OR ("2019 novel coronavirus") OR ("2019 novel coronavirus infection") OR (COVID19) OR ("coronavirus disease 2019") OR ("coronavirus disease-19") OR ("2019-nCoV disease ")OR ("2019 novel coronavirus disease") OR ("2019-nCoV infection") OR ("COVID-19 virus spike glycoprotein") OR ("2019-nCoV spike glycoprotein")
